# First Molar Necessary to Dentists’ Financial Success

**Published:** 1902-05-15

**Authors:** C. M. Wright

**Affiliations:** Cincinnati


### FIRST MOLAR NECESSARY TO DENTISTS' FINANCIAL SUCCESS.

BY C. M. WRIGHT, D.D.S., CINCINNATI.

Twenty years ago a dentist of fine address and education, of refined tastes, cultivated by study and travel, with a large practice among the creme de la creme of the city in which he was located, accompanied me to a dental society meeting, the first he had ever attended. The subject under consideration was, "Shall the Sixth Year Molar be Preserved?" Shortly before that time Dr. Atkinson had suggested, that if for any cause early in a child's life one of these molars had to be sacrificed, it might be well, in order to preserve harmonious facial development, to extract the remaining three teeth. The question was warmly discussed pro and con, and purely from scientific, surgical and esthetic standpoints. After the meeting, as my friend and I were discussing the menu at the hotel, I asked him if he had not found the meeting interesting and profitable. His reply gave food for thought as a broad business view. Said he, "I was disgusted with the whole affair. I never listened to such stuff. Figure it out for yourself. The sixth year molars are particularly liable to decay, and each one may develop five or more cavities on the different surfaces. Let us say five, then we may expect to be called upon to insert four times five fillings in each patient's sixth year molars. Each filling, we may say, will be worth $4. Four times twenty equals $80 for each patient's four first molars. Supposing a dentist has but one hundred patients each year who require these operations; one hundred times $80 equals $8,000. Supposing that there are 20,000 dentists in the world who have each these one hundred patients a year; multiply $8,000 by 20,000 equals $160,000,000, and you have that amount lost to the dental profession each year by the extraction of these sixth year molars. Bah! no wonder dentists are poor."

My friend retired from practice after twenty years of devotion to his work and lived ever afterwards like a gentleman.—*Digest.*

